# Supplementary material for: An open-source system for efficient clinical trial support: The COMET study experience
Source: PLoS One. 2023 Nov 27;18(11):e0293874. doi: 10.1371/journal.pone.0293874 (PMC10681164; doi:10.1371/journal.pone.0293874)
Supplement: S1 File — (PDF) [file pone.0293874.s001.pdf]

An Open-Source System for Efficient Clinical Trial Support: the COMET study experience

jclutton[1], evidoni[1], Dinesh Pal Mudaranthakam[1], Robert Neal Montgomery[1], Erin Blocker[2], Ashley Shaw[1], Amanda Szabo Reed[1]

[1]University of Kansas Medical Center; [2]Emporia State University

jclutton

Protocol Info: jclutton, evidoni, Dinesh Pal Mudaranthakam, Robert Neal Montgomery, Erin Blocker, Ashley Shaw, Amanda Szabo Reed . An Open-Source System for Efficient Clinical Trial Support: the COMET study experience. **protocols.io** https://protocols.io/view/an-open-source-system-for-efficient-clinical-trial-c4b2ysqe

Created: Oct 31, 2023

Last Modified: Nov 01, 2023

PROTOCOL integer ID: 90202

Keywords: comet, r, fitbit, clinical trial support, data infrastructure, informatics

DISCLAIMER

The authors make no guarantee of accuracy or compatibility with the user's system or project.

ABSTRACT

Exercise clinical trials are complex, logistically burdensome, and require a well-coordinated multi-disciplinary approach. Challenges include managing, curating, and reporting on many disparate information sources, while remaining responsive to a variety of stakeholders. The Combined Exercise Trial (COMET, NCT04848038) is a one-year comparison of three exercise modalities delivered in the community. Target enrollment is 280 individuals over 4 years. To support rigorous execution of COMET, the study team has developed a suite of scripts and dashboards to assist study stakeholders in each of their various functions. The result is a highly automated study system that preserves rigor, increases communication, and reduces staff burden. This manuscript describes system considerations and the COMET approach to data management and use, with a goal of encouraging further development and adaptation by other study teams in various fields.

Funders Acknowledgement:

National Institute on Aging
Grant ID: P30 AG072973

National Institute on Aging
Grant ID: RO1 AG070036

National Institute on Aging
Grant ID: K01 AG072034

Leo and Anne Albert Charitable Trust
Grant ID: N/A

GUIDELINES

This data infrastructure project was specially designed for the Combined Exercise Trial (https://doi.org/10.1016/j.cct.2022.106805); however, with some adaptations it can be applied to other studies. We suggest using the data flow diagram below and the paper to plan out what aspects of the data infrastructure you'd like to use.

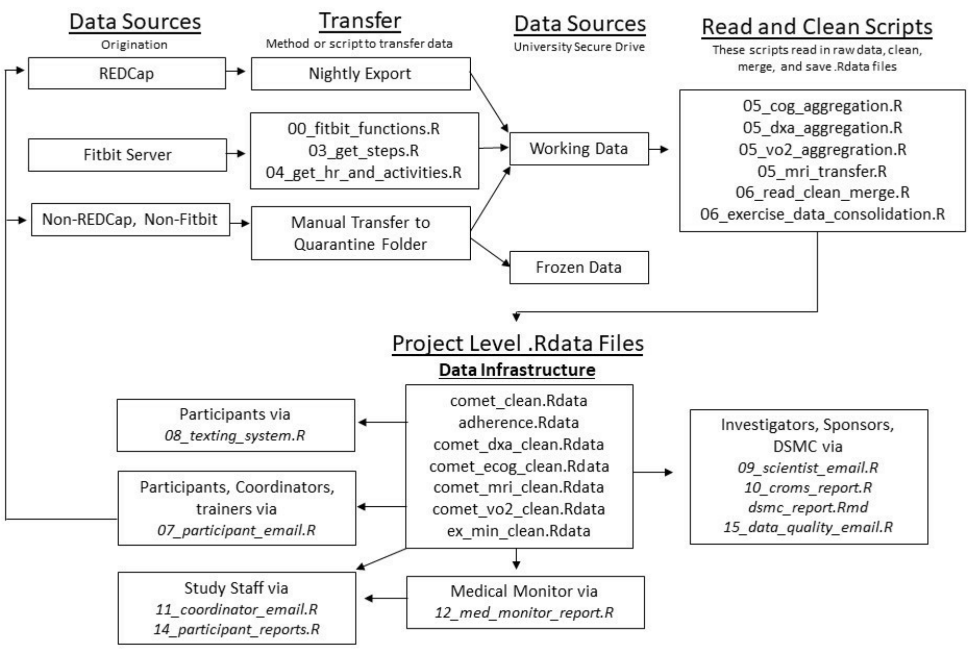

Figure 1: The COMET daily data infrastructure.

SAFETY WARNINGS

The code will not work automatically and will require a heavy amount of adaptation. The COMET study is underway until at least 2026; therefore the data and more importantly, the structure of the data are private. This will require some interpretation of the user to adapt. We hope to make the data public when possible.

ETHICS STATEMENT

This work was funded by grants from the National Institute on Aging P30 AG072973, R01 AG070036, K01 AG072034, and the Leo and Anne Albert Charitable Trust which do not necessarily endorse the statements. The content is solely the responsibility of the authors and does not necessarily represent the official views of the National Institutes of Health. The funders had no role in study design, data collection and analysis, decision to publish, or preparation of the manuscript.

BEFORE START INSTRUCTIONS

1. Be sure you have R downloaded (R: The R Project for Statistical Computing (r-project.org))

Software	
**R programming language**	NAME
The R Foundation	DEVELOPER
Comprehensive R Archive Network	SOURCE LINK

2. Be sure you have python downloaded (Download Python | Python.org)

Software	
**python**	NAME
3.6	OS
Guido van Rossum	DEVELOPER

3. We recommend using Rstudio as an R environment (Download RStudio - Posit)

Software	
**R Studio Desktop**	NAME
The R Studio, Inc.	DEVELOPER

Planning

1 Review the code (GitHub - cometstudy/OSSforEfficientClinicalTrialSupportCOMET) and plan what aspects of the project you'd like to adapt.
Some possibilities include:

- Fitbit data infrastructure
- DSMC reporting
- Study staff reporting
- Email modules
- Data storage
- Some approximation of the whole project

2 The code is designed to work in combination with a REDCap project. The data dictionary for the REDCap project can be found here: cometstudy/OSSforEfficientClinicalTrialSupportCOMET (github.com). We suggest getting a feel for the REDCap project before making decisions about what aspects of the code you'd like to adapt.
Note: Some surveys have been removed from the REDCap project, as they are available in the REDCap Instrument Library.

Operationalize

3 If you plan to use parts of the project that require REDCap instruments, i.e. output scripts:

1. Download the data dictionary from the GitHub project: cometstudy/OSSforEfficientClinicalTrialSupportCOMET (github.com)
2. Upload the data dictionary into your REDCap project.

If you plan to use parts of the project that require REDCap, but don't have access to REDCap, you may be able to approximate a similar system using other Electronic Data Capture or data storages systems.

4 Download the code: GitHub - cometstudy/OSSforEfficientClinicalTrialSupportCOMET

5 The project infrastructure runs every morning using the comet_nucleus.R script. This script can be used to get acquainted with the daily processes. The script may also be set to run in an automated fashion using a cronjob or similar system operation.

6 All names, emails, drives, and pathways have been scrubbed from the code. Be sure to correct them. A file with all scrubbed pathways is included.
Note: To find every instance of a text in a directory (i.e. a scrubbed pathway), you can use the ctrl+shift+f command in Rstudio.

scrubbed_pathways.xlsx

7 Adapt the project to fit your needs!
